# *Ex vivo* Gliadin Stimulation of Intestinal Cells

**DOI:** 10.1016/bs.mcb.2022.09.018

**Published:** 2022-10-27

**Authors:** Linda Zhang, Chuan He

**Affiliations:** 1 Department of Chemistry, Department of Biochemistry and Molecular Biology, Howard Hughes Medical Institute, University of Chicago, Chicago, Illinois, USA

**Keywords:** celiac disease, *in vitro*, gliadin stimulation, intestinal epithelial cell

## Abstract

Celiac disease is an autoimmune response to gluten proteins. While causes for celiac disease have been identified, there is no effective treatment other than diet control. In vitro models for celiac disease are important for quickly gaining understanding of the disease mechanism and testing potential treatments. Here we describe an *ex vivo* stimulation of intestinal epithelial cells with gliadin peptides as a method to induce celiac disease features *in vitro*.

## 1. Introduction

Celiac disease (CD) is an autoimmune disease triggered by the intake of gluten found in wheat, rye, and barley. Around 1% of the population have CD, and most of them express at least one of the two MHC II genes: HLA-DQ2 and HLA-DQ8 haplotypes (Caio et al., 2019). However, other non-HLA genes are also associated with CD (Trynka et al., 2011). Gluten activates innate and adaptive immune responses and induces the secretion of cytokines (Goel et al., 2019; Jelínková et al., 2004). IL-15 is a major pro-inflammatory cytokine elevated in both epithelial and lamina propria. IL-15 activates and expands intraepithelial lymphocytes (IELs) (Mention et al., 2003). Simultaneously, intestinal cells release autoantigen tissue transglutaminase (tTG) in response to gluten uptake. CD patients have villous atrophy and inflamed lamina propria (MacDonald et al., 1999). Diagnosis involves biopsy and detection of anti-TG2 IgG and IgA.

Despite the genetic and immune system factors affecting celiac disease have been identified, there is no effective treatment to prevent or reverse CD. CD patients are limited to life-long diet control. The lack of treatment is in part due to the lack of reliable animal models that fully capture the disease features of patients. Early animal models involve feeding pups with a gluten-containing diet. These spontaneous models with dogs and monkeys are sensitive to gluten and show villous atrophy and intraepithelial lymphocytes. However, their disease is not linked to MHC class II genes, and they do not develop gliadin or TG2 antibodies (Bethune et al., 2008; Hall et al., 1992; Polvi et al., 1998). Non-obese diabetic (NOD) mice are sensitive to a gluten diet, leading to villous atrophy and increased IELs, but failed to represent the antibody response to tTG (Sblattero et al., 2005).

Early transgenic mouse models failed to capture the full characteristics of CD. For example, mice with HLA gene expression have elevated cytokine secretion and gliadin antibody production in response to gluten, but fail to develop enteropathy (Black et al., 2002). Recently, the Jabri Lab established the first successful mouse model for celiac disease (Abadie et al., 2020). Mice expressing HLA-DQ8, a genetic variant found in all celiac disease patients, with overexpression of IL-15 in lamina propria and intestinal epithelial cells develops villous atrophy in response to gluten diet, which is reversed after gluten-free diet.

Mouse models are time-consuming and expensive. *In vitro* models provide a time- and cost-effective alternative for understanding the mechanism and testing treatments for celiac disease. Colon epithelial-derived cell lines serve as a simple *in vitro* model. Using intestine cell line Caco-2, the mechanism by which gluten affects intestine cells was studied, and potential treatments for alleviating gluten-induced inflammatory response were also tested (Kramer et al., 2019; Rivabene et al., 1999). These cell lines derived from tumor tissues do not have the same genetic variants as celiac disease patients. However, cell lines could potentially be engineered with genome editing technologies. This cell model has the advantage of studying a specific type of cells but lacks the interactive information between the intestine and immune cells. Alternatively, primary cells can be isolated from animal tissues or patient biopsies. Patient biopsies are more representitve of the disease, but sample is often limited. Organ-on-a-chip is a powerful in vitro model that can mimic the *in vivo* organ environment. This system includes microfluidic forces that can be used to simulate the intestine environment. A gut-on-chip model with Caco-2 cells generated a small section of 3D intestine that has functional properties of the human intestine (Kim et al., 2012).These could serve as valuable *in vitro* CD models with the appropriate stimulation. However, unlike animal models where gluten diets can be fed to induce disease symptoms, these systems lack enzymatic activities to digest gluten. It is known that gluten is digested into smaller proteins gliadins and glutenins, with gliadin being the main disease-causing agent (Arentz-Hansen et al., 2002).Therefore, *in vitro* systems rely on stimulation by gliadins.

**Fig. 1.**
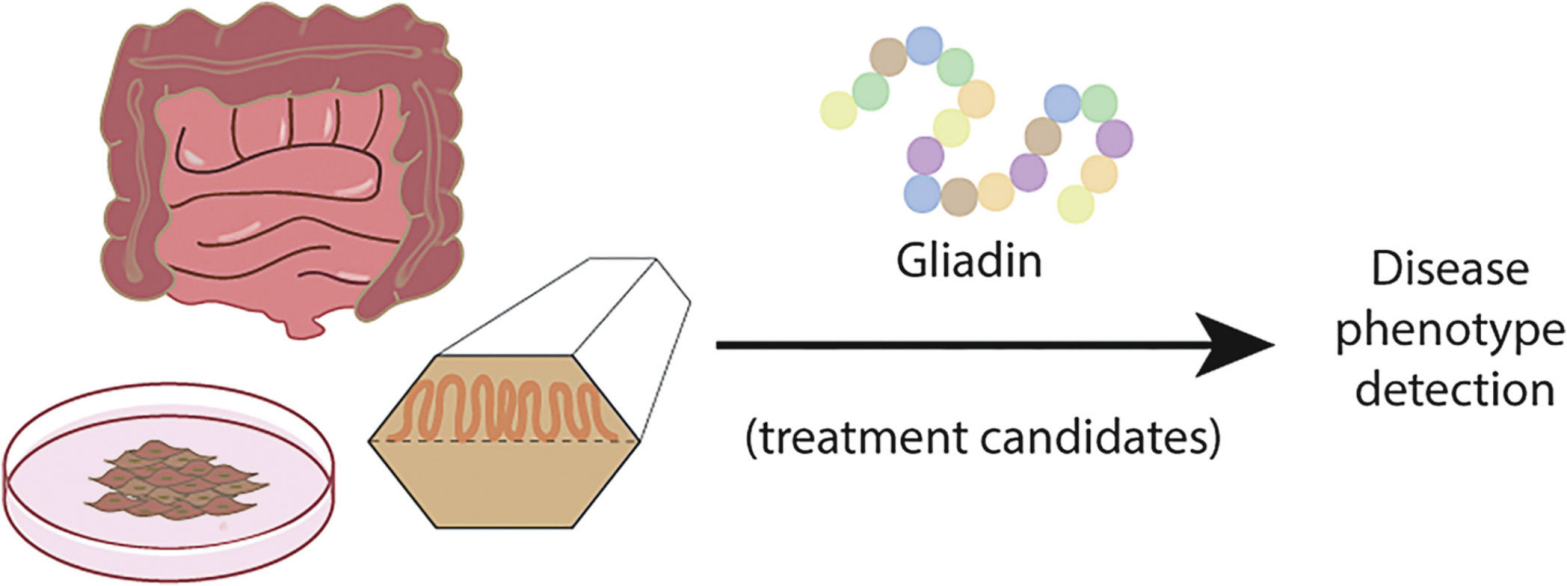
Stimulation of tissue biopsies, cultured cells, or gut-on-chip with gliadin peptides to induce celiac disease phenotypes. The system can be adapted to test treatment options for celiac disease.

Here we describe an *in vitro* stimulation of intestine epithelial cells with gliadin (Fig. 1). This protocol could be adapted to study cell lines, cells isolated from mouse tissues or patient biopsies, or gut-on-chip models, enabling *in vitro* studies of CD.

## 2. Reagents

4% sodium hypochlorite

PBS

Digestion buffer:

1.2 mM KH_2_PO_4_
680 mM Na_2_HPO_4_
2.7 mM KCl
150 mM NaCl
1.5 mM EDTA

1 M DTT

Pepsin Trypsin digested gliadin (PTG)

DMEM media with 10% FBS and penicillin-streptomycin

## 3. Mouse intestinal epithelial cell extraction

1. Before starting, buffer should be cooled and kept on ice (see Note 1).
2. Euthanize mouse and harvest the intestine. Lay the mice on its back and spray the abdomen with 70% ethanol. Open the abdomen and lightly pull the intestine out with forceps. Carefully cut the tissues and fat connecting adjacent regions to release the intestine. The intestine starts below the stomach and ends before the cecum. Starting from the end closest to the stomach, the intestine can be sectioned into the duodenum, jejunum, and ileum (see Note 2).
3. Cut the intestine by region of interest. Store the organ in a dish with cold PBS.
4. Holding the intestine with forceps, cut it open lengthwise with scissors (see Note 3).
5. Place the intestine in ice-cold PBS, swirl around to remove any excrement. Replace PBS and repeat until clean (see Note 4).
6. Prepared 0.04% sodium hypochlorite in PBS (see Note 5).
7. Place clean intestine in 50 mL sodium hypochlorite and incubate 15 minutes on ice (see Note 6).
8. Remove pieces from sodium hypochlorite solution and rinse with PBS (see Note 7).
9. Add DTT to digestion buffer to 0.5 mM (see Note 8).
10. Place intestine pieces in 5 mL digestion buffer in a falcon tube.
11. incubate on ice for 15 minutes.
12. Carefully pour out digestion buffer, leaving the intestine pieces inside, then add 5 mL PBS.
13. Vortex for 15 seconds (see Note 9).
14. Use forceps to remove intestine pieces. The solution now contains epithelial cells (see Note 10).
15. Spin down the epithelial cells containing solutions at 300 × g for 3 minutes (see Note 11).

## 4. Gliadin stimulation

**Fig. 2.**
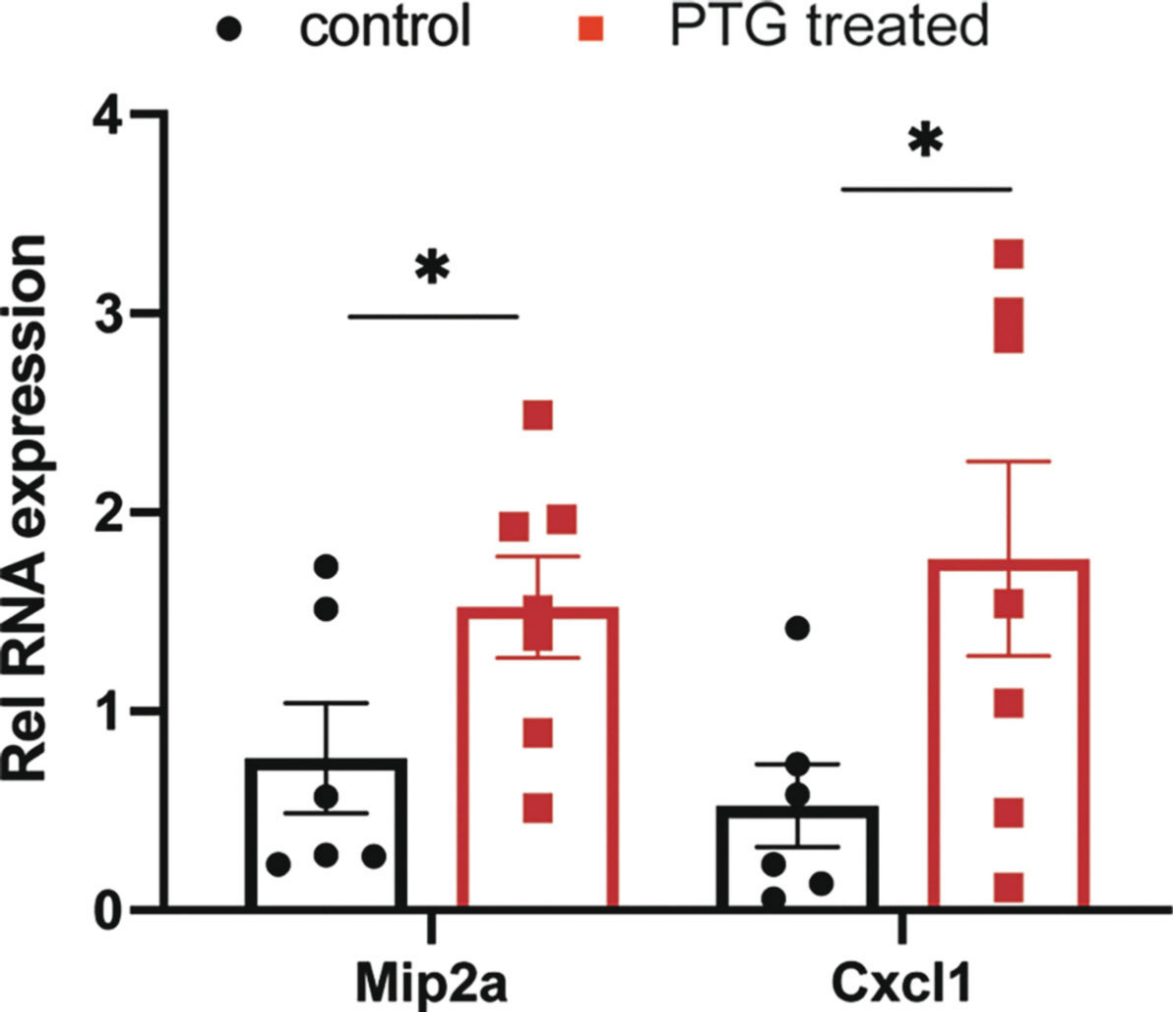
RT-qPCR of IL8 mouse homologs Mip2a and Cxcl1 with control and PTG stimulated samples.

1. Resuspend cells with DMEM media, or media containing 250 μg/mL PTG. Place cells in cell culture plates (see Note 12-15).
2. Incubate cells at 37°C for 4 hours (see Note 16-17).
3. Collect cells by pipetting to resuspend all cells, spin down cells and remove supernatant (see Note 18).
4. Resuspend cell pellet with lysis buffer with protease inhibitor for western blotting or with Trizol for RNA extraction. Celiac disease indicators can be examined. For example, one feature of celiac disease is the secretion of IL8 by enterocytes. We performed RT-qPCR with PTG stimulated or non-treated samples, and observed a two-fold increase in Mip2a and Cxcl1, which are the mouse functional homologs of IL8 (Fig. 2).

## 5. Concluding remarks

Recent advances in animal models provide a valuable system for developing and testing new treatments for celiac disease. However, *in vitro* models have a shorter turnover time, which is essential for drug development and optimization. Cell lines, animal tissues, patient biopsies, and organ-on-chip can be used for studying the gut *in vitro*. To study celiac disease using these systems, cells or organs must be stimulated with gliadin to trigger disease phenotypes. We provided a method for inducing celiac disease features by stimulation with gliadin. This protocol uses freshly isolated mouse intestinal cells as an example. However, the sample preparation could be modified to isolate a specific cell population, cell lines, patient biopsies, or organ-on-chip. Aided by this method, a new mechanism of celiac disease was discovered (Olazagoitia-Garmendia et al., 2022).

The use of freshly isolated tissue samples has the limitation of proliferative ability when compared to cell lines. Furthermore, the use of specific cell lines reveals cell type-specific disease mechanisms. However, studies using whole tissue biopsies provide more complex interactions between different cell types. Tissues also allow for the simultaneous studies of different cell types. Patient biopsies has also been used as a more accurate system to study disease mechanism (Fernandez-jimenez et al., 2014; Hüe et al., 2004; Maiuri et al., 2003). However, patient biopsies are often scarce. While mouse models cannot fully represent human celiac disease, they provide a platform for probing experiment conditions before studying human samples.

We chose C57BL/6J mice because this strain is easily accessible. The protocol can be adapted for other mouse strains such as the recently established DQ8-D^d^-villin-IL-15tg strain. This system could also serve as the first screening of new treatments, and the most effective ones could be further validated with the more accurate transgenic mouse or patient samples, reducing time and resources in testing new drugs.

## Notes

1. Although not required, it’s suggested to sterilize buffer and process samples in a biosafety hood, especially for longer culture of epithelial cells.
2. C57BL/6J mice were used at the age of 4 weeks old. Mice were euthanized by CO_2_ asphyxiation. Other strains of mice could be used.
3. Intestine can also be flushed with PBS and an insulin syringe.
4. After the last wash, tissue can be stored in PBS on ice until all samples are ready for the next step.
5. Dilute 4% sodium hypochlorite 100-fold. For each sample, add 0.5 mL 4% sodium hypochlorite into 50 mL PBS.
6. This step removes gut bacteria.
7. Place tissue in copious amount of PBS to remove remaining hypochlorite solution.
8. DTT should be freshly added before use.
9. Samples should be vortexed vigorously to mechanically detach loose cells.
10. This yields thin sheets of epithelial cells that will attached to cell culture plates. To obtain more cells, use the tissue from step 14 to repeat steps 10–14 two more times.
11. To further obtain single cell suspension, cells can be treated with 0.25% Trypsin for 3–5 minutes followed by pipetting. Then add 3–5 volumes of DMEM to quench enzymatic activities. Spin down cells 300 × g for 3 minutes.
12. Specific cell population could be further isolated.
13. Sheets of epithelial cells cannot be counted accurately. Resuspend and plate cells so that cells form a mono layer at the bottom. Dilute if needed.
14. Concentration of GTP stock was determined with the Pierce BCA Protein Assay Kit with BSA standards. Samples in DMEM without GTP serves as unstimulated controls.
15. To test treatments, isolated cells can be pretreated prior to gliadin stimulation, or stimulated in the presence of treatments.
16. This specific gliadin treatment dose and duration was found to be effective for cell lines and human and mouse biopsies (Fernandez-jimenez et al., 2014; Olazagoitia-Garmendia et al., 2022). However, these conditions could be optimized for different samples and purpose.
17. For longer culture, additives such as insulin and EGF might be added to sustain cell viability.
18. Cells do not attach tightly to the culture dish after short incubation. Long incubation time might cause cells to attach more firmly. Use a cell lifter if needed.
